# Supplementary material for: A pilot study for whole proteome tagging in Caenorhabditis elegans
Source: eLife. 2026 Jun 24;15:RP110717. doi: 10.7554/eLife.110717 (PMC13293606; doi:10.7554/eLife.110717)
Supplement: Peer review history

# Editor report: eLife Assessment

**DOI:** 10.7554/eLife.110717.4.sa0

**Authors:** Douglas Portman

**Affiliations:** University of Rochester United States

**Keywords:** Important, Solid

The nematode *C. elegans* is an ideal model in which to achieve the ambitious goal of having a genome-wide atlas of protein expression and localization. In this paper, the authors develop a rational strategy for at-scale tagging of all protein coding genes with fluorescent markers, providing **solid** evidence that it would be a feasible foundation for a community-based, genome-wide effort. This work should serve as an **important** springboard for discussions about how to achieve this worthy and impactful goal.

# Referee report: Reviewer #1 (Public review)

**DOI:** 10.7554/eLife.110717.4.sa1

Summary:

Eroglu and Hobert demonstrate that injecting CRISPR guides and repair constructs to target three genes at a time, tagging each with a different fluorescent protein, and selecting which gene to tag with which fluorophore based on genes' expression levels, can improve efficiency of gene tagging.

Strengths:

This manuscript demonstrates that three genes can be targeted efficiently with three different fluorophores. It also presents some practical considerations, like using the fluorophore least complicated by agar/worm autofluorescence for genes with low expression levels, and cost calculations if the same methods were used on all genes.

Weaknesses:

Eroglu has demonstrated in a previous publication that single-stranded DNA injection can increase efficiency of CRISPR in *C. elegans*, while inserting two fluorescent proteins and a co-CRISPR marker into three loci, and Paix et al 2015 demonstrated simultaneous insertion of two fluorescent tags. The current work is valuable and incremental advance. In general, I applaud the authors' willingness to strategize about how whole proteome tagging might be accomplished. I predict that the advance here will be one of many small advances that will get the field to that goal. The title oversells the advance presented, in my view, since seems like one among many key advances, and the first sentence of the Discussion seems a more apt summary of the key advance here.

Some injections targeted genes on the same chromosome together, which will create unnecessary issues when doing crossing that will be useful for some future experiments. This made me wonder if injecting 3 together really is helpful vs targeting each gene separately, since only 5 worms need to be injected. It cuts time down by 2/3, but perhaps avoiding targeting the same chromosome with two tags would be useful.

The limited utility of current blue fluorescent proteins makes me wonder if it's worth using at this stage, before there are better blue fluorescent proteins, or better yet, far red, to avoid issues with live imaging under phototoxic UV or near-UV illumination.

# Referee report: Reviewer #2 (Public review)

**DOI:** 10.7554/eLife.110717.4.sa2

Overall, we found the responses to be quite recalcitrant.

We have one remaining composite concern about the comparison between observed expression patterns with the new strains versus published data.

First, the authors only report patterns for one stage while it should be not too much effort to image the different life stages. However, since this is a revision, we are not formally requesting they do this.

Second, in the now provided Table (thank you) 'observed expression' (last column) is lacking for 9 of the 30 proteins, and for 6 of these the procedure was not successful. Why not report patterns for the other three? It is confusing also because on page 5, the authors say that "overall, 24 of 30 tags ...all of which were visible with fluorescence stereomicroscopy" - are we missing something? Also, they then said that they "obtained 6/9 of the originally failed tags"; why are the corresponding patterns not included in table 1, and are 9 proteins still labeled as "no" in the "success?" Column?

Third, we strongly feel that the response to our comments about expression patterns is not adequate. On page 5 the authors say that "all proteins were expected to be ubiquitously expressed" and that "scRNA-seq indicated that transcript abundance was ubiquitous and without strong tissue-specific enrichment with few exceptions". However, in their rebuttal, the authors now argue for tissue-specific expression for proteins with paralogs, turning around their own argument! Moreover, their Table indicates that many genes show tissue-enriched expression by RNA-seq while many of their tagged proteins exhibit ubiquitous expression.

Overall, this indicates that both the overall accomplishment of generating tagged protein strains and analyzing their expression is oversold.

# Referee report: Reviewer #3 (Public review)

**DOI:** 10.7554/eLife.110717.4.sa3

Summary:

The authors argue that establishing the expression pattern and sub-cellular localisation of an animal's proteome will highlight hypotheses for further study. This claim is probably accepted by many in the community. This manuscript seeks to confirm the feasibility of establishing such a resource, by using current transgenic methods to knock in DNA encoding different colored fluorescent tags into *C. elegans* genes.

Strengths:

The authors make the points above. For example, they provide evidence that the *C. elegans* germline harbors two populations of mitochondria that differ qualitatively in the proteins they express. They also confirm that labelling the whole proteome is an achievable goal with relatively limited resources and time.

Weaknesses:

The work is somewhat incremental in that it uses existing transgenic technology. Cell biology in *C. elegans* is challenging because of the small size of many of its cells, notably neurons. This can make establishing the sub-cellular localisation of a fluorescently tagged protein, or co-localizing it with another protein, tricky. The authors point out in their introduction that advances in light microscopy such as diSPIM, STED and ISM (a close relative of SIM), have increased the resolution of light microscopy. They also point out that recent advances in expansion microscopy can similarly help overcome the resolution limit. However, they do not use these technologies to characterize their transgenic strains.

# Referee report: Reviewer #4 (Public review)

**DOI:** 10.7554/eLife.110717.4.sa4

Summary:

Tagging the entire proteome of a metazoan would be a landmark achievement, providing a powerful complement and extension to existing "omic" catalogs in model systems. Here, Eroglu and Hobert argue that efficiently tagging multiple loci in a single "batch" would make the community-based achievement of this goal realistic. They provide rigorous evidence that such an approach is indeed feasible, exploring issues related to efficiency, design and screening strategies, disruption of gene function, and the potential for endogenously tagged alleles to reveal unexpected aspects of protein expression and localization. While the work has some minor gaps that are important to rigorously assess the feasibility of the proposed effort, the detailed and valuable insights that emerge should provide impetus to the community to coordinate efforts to make this ambitious goal a reality.

Strengths:

The work has numerous strengths. The authors provide compelling evidence that:

- three distinct loci can be efficiently targeted with three distinct fluorescent tags in a single injection.

- thoughtful targeting design can reduce the likelihood of disruption of function by the tag.

- systematic design principles based on expression level and predicted localization/function can be used to optimize tagging strategies.

- the resulting tags can provide unexpected insight into patterns of protein production and subcellular localization.

Not all of these advances are novel in themselves, but taken together, they represent an important technical and conceptual advance. The most important strength comes from the exceptionally high value of the goal itself, in that the work is that it has the potential to spur a community-wide effort toward achieving the ambitious goal of proteome-wide tagging.

Weaknesses:

The work's shortcomings are minor.

- One concern has to do with the feasibility of the proposed screening strategies. The experimental design cleverly coinjects tags for three loci in different gene expression 'zones'; this expression level determines which tag will be used. As the authors allude to, there is an important distinction between genes with the same overall FKPM value between those that are expressed broadly and those focally expressed in a specific tissue. The proposed strategy claims that there are a sufficient number of highly expressed genes "to be used as visible markers" for recovering successfully edited animals. It would be useful for the authors to discuss the issue of broad vs focused expression among this set of genes a bit more thoroughly, with an eye toward the issue of how likely it is that these genes could indeed consistently be used as visible markers, particularly for those at the low end of this limit.

- What fraction of the proteome (on a per-gene basis) is secreted proteins? How difficult will it be to screen these for successful tags? Are there specific tags that would be more optimal for secreted proteins? (The authors mention the use of an SL2 or T2A cassette to label the cells in which these proteins are expressed but note that there are technical challenges associated with doing this at scale.)

- For secreted and/or weakly expressed genes, it would be useful for the authors to estimate for what fraction of these would successful insertions need to be screened by PCR, and what resources (time and money) this would likely entail.

- For how many genes would a single tag not capture all predicted isoforms?

- Finally, some readers might object to the authors' assertion in the abstract that this work is "a first step in this direction" (presumably referring to designing a strategy for whole-proteome tagging). There is no concern that the authors are disregarding the extensive work of other groups, as they explicitly mention the contributions of other groups to the foundation that enables the present work. However, the spirit of the abstract could be misinterpreted by a well-intentioned reader.

# Author comment: Author response

**DOI:** 10.7554/eLife.110717.4.sa5

**Authors:** Matthew Eroglu, Oliver Hobert

**Affiliations:** Columbia University New York United States; Howard Hughes Medical Institute New York United States

The following is the authors’ response to the previous reviews

**Reviewer #1 (Public review):**

Summary:

Eroglu and Hobert demonstrate that injecting CRISPR guides and repair constructs to target three genes at a time, tagging each with a different fluorescent protein, and selecting which gene to tag with which fluorophore based on genes' expression levels, can improve efficiency of gene tagging.

Strengths:

This manuscript demonstrates that three genes can be targeted efficiently with three different fluorophores. It also presents some practical considerations, like using the fluorophore least complicated by agar/worm autofluorescence for genes with low expression levels, and cost calculations if the same methods were used on all genes.

Weaknesses:

Eroglu has demonstrated in a previous publication that single-stranded DNA injection can increase efficiency of CRISPR in *C. elegans*, while inserting two fluorescent proteins and a co-CRISPR marker into three loci, and Paix et al 2015 demonstrated simultaneous insertion of two fluorescent tags. The current work is valuable and incremental advance. In general, I applaud the authors' willingness to strategize about how whole proteome tagging might be accomplished. I predict that the advance here will be one of many small advances that will get the field to that goal. The title oversells the advance presented, in my view, since seems like one among many key advances, and the first sentence of the Discussion seems a more apt summary of the key advance here.

Some injections targeted genes on the same chromosome together, which will create unnecessary issues when doing crossing that will be useful for some future experiments. This made me wonder if injecting 3 together really is helpful vs targeting each gene separately, since only 5 worms need to be injected. It cuts time down by 2/3, but perhaps avoiding targeting the same chromosome with two tags would be useful.

The limited utility of current blue fluorescent proteins makes me wonder if it's worth using at this stage, before there are better blue fluorescent proteins, or better yet, far red, to avoid issues with live imaging under phototoxic UV or near-UV illumination.

These comments are a repeat of the original comments, and we refer the reader to our response to the original comments.

**Reviewer #2 (Public review):**

Original Review:

The manuscript by Eroglu and Hobert presents a set of strains each harboring up to three fluorescently tagged endogenous proteins. While there is technically nothing wrong with the method and the images are beautiful, we struggled to appreciate the advance of this work - who is this paper for?

As a technical method, the advance is minimal since the first author had already demonstrated that three mutations (fluorophore insertion and co-CRISPR marker) could be introduced simultaneously.

As a pilot for creating genome-scale resources, it is not clear whether three different fluorophores in one animal, while elegantly designed and implemented, will be desired by the broader community.

Finally, the interpretation of the patterns observed in the created lines leaves much to be desired. A Table with all the observations must be included and can replace the tedious (and often wrong) descriptions of the observations with the different lines. It would be too much to point out every mistaken expectation of protein expression. Two examples include:

The expectation that ACDH-10 is enriched in the intestine and epidermal tissues (hypodermis) is naïve - there are multiple paralogs of this protein (look at WormPaths or WormFlux) that may share functions in different tissues. There is also no reason to assume that fatty acid metabolism does not occur in other tissues (including the germline). Finally, there are no published studies about this enzyme, so we really don't know for sure what it's doing.

The expectation that HXK-1 is ubiquitously expressed is similarly naïve. There are three paralogous enzymes that are all associated with the same reaction, and we have shown that these three function redundantly in vivo, perhaps in different tissues (PMID: 40011787). Moreover, single cell RNA-seq data (PMID: 38816550) also shows enrichment of hxk-1 in gonadal sheath cells.

The table should have at least the following information: gene/protein name - Wormbase ID - TPM levels of single cell data assigned to tissues for L2, L4 and adult (all published) - tissues in which expression is observed in the lines presented by the authors.

Other points:

(1) We would encourage the authors to provide systematic validation of the reported insertions. The manuscript reports that 24 of 30 tags were isolated and visible but does not clearly state whether each isolated line was confirmed by sequence‑level validation to be correctly in‑frame and free of unintended mutations at the target locus.

(2) The manuscript presents aggregated success counts (e.g., 8/10 mTagBFP2 tags, 9/10 mStayGold, 7/10 mScarlet3) and useful narrative descriptions of injection outcomes. We suggest also to include per‑locus success rates.

(3) For pools that required re‑injection after initial failures, we would like to see a description of the specific changes that were made to the injection mixes or procedures (e.g., new repair template prep, different Cas9 reagent lot, guide redesign). This will be useful troubleshooting information for others.

(4) The authors states that the fluorophore sequences are codon-optimized for *C. elegans*. We suggest they provide the exact donor/tag sequences used specifically state whether the fluorophore sequences contain any synthetic/artificial introns or other sequence modifications (e.g., silent PAM‑disrupting mutations) were included in the donor templates.

(5) Page 3: Include a reference for "The *C. elegans* genome encodes around 20,000 genes"

We hope these comments are useful.

Comments on Revised Version:

Overall, we found the responses to be quite recalcitrant.

We have one remaining composite concern about the comparison between observed expression patterns with the new strains versus published data.

First, the authors only report patterns for one stage while it should be not too much effort to image the different life stages. However, since this is a revision, we are not formally requesting they do this.

Second, in the now provided Table (thank you) 'observed expression' (last column) is lacking for 9 of the 30 proteins, and for 6 of these the procedure was not successful. Why not report patterns for the other three? It is confusing also because on page 5, the authors say that "overall, 24 of 30 tags ...all of which were visible with fluorescence stereomicroscopy" - are we missing something? Also, they then said that they "obtained 6/9 of the originally failed tags"; why are the corresponding patterns not included in table 1, and are 9 proteins still labeled as "no" in the "success?" Column?

We appreciate the chance to clarify this matter: There are only 6 “no” in the “success” column. In two cases, HAT-1 and CBP-1, expression was dim at F1 but still sufficient to pick positive worms and quantify success rate at the locus. We noted these as “dim” on the table to indicate that if expression was lower, we likely would not have been able to isolate them at F1. In one case, COX-6B, expression was too dim at F1 to be isolated but was sufficient at F2 to be visualized and isolated from parents that were positive for the other two tags. We now clarified this distinction in the table and accompanying text: “Fluorescent signals of HAT-1::mScarlet3 and CBP-1::mScarlet3 in F1 progeny were dim but still sufficiently visible for quantification of knock-in efficiency, indicating that they are at the lower end of detectability for mScarlet3.”

We imaged worms that had multiple tags as proof of principle and are happy to provide strains to those who would like to image/study them. At this point we are not convinced that imaging more worms would add to the conceptual framework.

Third, we strongly feel that the response to our comments about expression patterns is not adequate. On page 5 the authors say that "all proteins were expected to be ubiquitously expressed" and that "scRNA-seq indicated that transcript abundance was ubiquitous and without strong tissue-specific enrichment with few exceptions". However, in their rebuttal, the authors now argue for tissue-specific expression for proteins with paralogs, turning around their own argument! Moreover, their Table indicates that many genes show tissue-enriched expression by RNA-seq while many of their tagged proteins exhibit ubiquitous expression.

We respectfully disagree that there is contradiction. In our response, the discussion on paralogs was added as a clarification in response to the referee’s original comments (e.g., regarding ACDH-10): “There is also no reason to assume that fatty acid metabolism does not occur in other tissues (including the germline).” We wanted to make it clear that we were not concluding fatty acid metabolism (or other processes) does not occur in other tissues.

We wish to stress that we never argued that paralogs could not fulfil the same essential function across tissues. The proteins were selected because their biological functions (e.g., glycolysis, fatty acid β-oxidation, translation) are broadly required, and that scRNA seq generally predicted broad expression with few exceptions as detailed in the text. Paralogs with similar activities (e.g., *hxk-1*, *-2*, *-3*) may overlap broadly in expression, or individual paralogs may carry out the process in different tissues provided one carries out the reaction in each tissue. For *acdh-10* and *hxk-1* specifically, both appear broadly expressed across tissues by scRNA-seq, with no consistent enrichment or depletion across datasets. So, our central point is that: for a specific gene involved in an essential process, transcript data alone are not sufficient to accurately predict tissue specific enrichment. Not that the processes do not occur in tissues where one paralog is absent. The possibility that a paralog may compensate for lack of expression is in no way contradictory with our conclusion.

The table does not generally show tissue-enriched expression: it simply lists three tissues with the highest quantitative value in the respective dataset. For instance, taking the first gene from the list (*Y82E9BR.3*) and looking at the Ghaddar dataset, the top 3 tissues (log2(TPM)) are: pharyngeal muscle (13.4), gonadal sheath (12.9), marginal cells (12.9). The next 3 tissues are: body wall muscle (12.9), pharyngeal epithelium (12.8), and intestine (12.3). Even when there were apparent enrichments among the top 3 tissues, there were significant disagreements between datasets, and beyond top 3 even greater disagreements (the datasets agreed on the top tissue only 4 times over the 30 genes). These indicate that much of the variation is attributable to experimental noise rather than true predicted enrichment. The referee points to HXK-1 being correctly gonadal sheath enriched in one scRNA dataset; however, the other two datasets actually show different sites as being highest, and the same dataset misses effects in other cases. This is precisely why protein level data is needed.

We further clarified this issue in the text: “We thus selected 30 genes across a variety of bulk transcript expression ranges which are generally predicted to be broadly expressed based on molecular function or, where molecular function was unknown (e.g., ZK632.9), single cell RNA sequencing (scRNA-seq) data (Table 1, Fig. 2A, B) (Gao et al., 2024; Ghaddar et al., 2023; Taylor et al., 2021).”

Overall, this indicates that both the overall accomplishment of generating tagged protein strains and analyzing their expression is oversold.

We have tried to make clear that our contribution is not a handful of new tagged strains added to the many that already exist. Rather, as stated in the abstract and elsewhere, we propose a strategy and provide proof-of-concept for scaling up tagging efforts. We believe the importance of this cannot be oversold.

**Reviewer #3 (Public review):**

Summary:

The authors argue that establishing the expression pattern and sub-cellular localisation of an animal's proteome will highlight hypotheses for further study. This claim is probably accepted by many in the community. This manuscript seeks to confirm the feasibility of establishing such a resource, by using current transgenic methods to knock in DNA encoding different colored fluorescent tags into *C. elegans* genes.

Strengths:

The authors make the points above. For example, they provide evidence that the *C. elegans* germline harbors two populations of mitochondria that differ qualitatively in the proteins they express. They also confirm that labelling the whole proteome is an achievable goal with relatively limited resources and time.

Weaknesses:

The work is somewhat incremental in that it uses existing transgenic technology. Cell biology in *C. elegans* is challenging because of the small size of many of its cells, notably neurons. This can make establishing the sub-cellular localisation of a fluorescently tagged protein, or co-localizing it with another protein, tricky. The authors point out in their introduction that advances in light microscopy such as diSPIM, STED and ISM (a close relative of SIM), have increased the resolution of light microscopy. They also point out that recent advances in expansion microscopy can similarly help overcome the resolution limit. However, they do not use these technologies to characterize their transgenic strains.

**Reviewer #4 (Public review):**

Summary:

Tagging the entire proteome of a metazoan would be a landmark achievement, providing a powerful complement and extension to existing "omic" catalogs in model systems. Here, Eroglu and Hobert argue that efficiently tagging multiple loci in a single "batch" would make the community-based achievement of this goal realistic. They provide rigorous evidence that such an approach is indeed feasible, exploring issues related to efficiency, design and screening strategies, disruption of gene function, and the potential for endogenously tagged alleles to reveal unexpected aspects of protein expression and localization. While the work has some minor gaps that are important to rigorously assess the feasibility of the proposed effort, the detailed and valuable insights that emerge should provide impetus to the community to coordinate efforts to make this ambitious goal a reality.

Strengths:

The work has numerous strengths. The authors provide compelling evidence that:

Three distinct loci can be efficiently targeted with three distinct fluorescent tags in a single injection.

Thoughtful targeting design can reduce the likelihood of disruption of function by the tag.

Systematic design principles based on expression level and predicted localization/function can be used to optimize tagging strategies.

The resulting tags can provide unexpected insight into patterns of protein production and subcellular localization.

Not all of these advances are novel in themselves, but taken together, they represent an important technical and conceptual advance. The most important strength comes from the exceptionally high value of the goal itself, in that the work is that it has the potential to spur a community-wide effort toward achieving the ambitious goal of proteome-wide tagging.

We appreciate the referee’s enthusiasm and hope that this will engage members of the community in a collective effort.

Weaknesses:

The work's shortcomings are minor.

One concern has to do with the feasibility of the proposed screening strategies. The experimental design cleverly coinjects tags for three loci in different gene expression 'zones'; this expression level determines which tag will be used. As the authors allude to, there is an important distinction between genes with the same overall FKPM value between those that are expressed broadly and those focally expressed in a specific tissue. The proposed strategy claims that there are a sufficient number of highly expressed genes "to be used as visible markers" for recovering successfully edited animals. It would be useful for the authors to discuss the issue of broad vs focused expression among this set of genes a bit more thoroughly, with an eye toward the issue of how likely it is that these genes could indeed consistently be used as visible markers, particularly for those at the low end of this limit.

To give two examples, this principle aided us with screening F54C8.1 and HAT-1. We added additional discussion on this to the first paragraph of the discussion: “For instance, we could clearly visualize F54C8.1::mScarlet3 in adult sperm by fluorescence stereomicroscopy despite a bulk FPKM of 16. Similarly, nuclear localized proteins will likely be easier to detect even at low expression levels, given the concentration of signal in small subcellular compartments. Indeed, this helped us detect HAT-1::mScarlet3 (56 bulk FPKM), which may have been too dim if distributed more broadly within cells.”

What fraction of the proteome (on a per-gene basis) is secreted proteins? How difficult will it be to screen these for successful tags? Are there specific tags that would be more optimal for secreted proteins? (The authors mention the use of an SL2 or T2A cassette to label the cells in which these proteins are expressed but note that there are technical challenges associated with doing this at scale.)

We added some of these points to the discussion: “Moreover, around 17% of the *C. elegans* genome (3,484 genes) may encode for secreted proteins (Suh and Hutter, 2012). Endogenous tagging of a substantial fraction of these proteins could reveal spatial patterns of secretion, distinguishing components that remain near their cell of origin from those that disperse to distal sites (Keeley et al., 2020). Tagging secreted proteins can also reveal sites of secretion – such as apical or basolateral membranes, or neurites – as has been observed for specific insulins (Sural et al., 2025) and for neuropeptides that localize selectively to synaptic regions (Toker et al., 2025).”

Various tags have been used for secreted proteins including Venus, TagRFP, and mNeonGreen. The pH of secretory vesicles is ~5.0-5.5, so chosen FPs should have a pKa below this range to avoid denaturation. All 3 fluorophores used here (mStayGold, mScarlet3 and mTagBFP2) have pKa’s below this range and would likely be fluorescent within secretory vesicles.

For secreted and/or weakly expressed genes, it would be useful for the authors to estimate for what fraction of these would successful insertions need to be screened by PCR, and what resources (time and money) this would likely entail.

We think that the bulk of ECM proteins would likely be visualizable without PCR due to their broad and stable expression, and as mentioned a good portion of these have been already tagged. However, it is likely that most of the secreted small peptides will have to be screened by PCR. We use homemade Taq, which makes material cost of the reagents minimal. A pair of genotyping primers costs ~$8 (~$27,872 for all secreted genes).

Hands on time for lysis of 48-96 worms is approximately 20-30 minutes, with time to set up PCR around 5-10 minutes per target, and time to load a gel of 10 mins. In a given pool, 2/3 could be a putative secreted protein; thus, the same lysed population would enable screening for two targets at once. Collectively, around 40-60 mins of hands-on time would be required for two genes (around 20-30 mins per gene). Given 18 targets are injected per day, if 12 are screened by PCR, the screening could be done in 6 hours per day without affecting throughput. Most of the time spent on PCR would be replacing fluorescence screening time and would not overlap with the rate limiting injection step, performed by a separate specialist.

For how many genes would a single tag not capture all predicted isoforms?

Around 25% of *C. elegans* genes are thought to undergo alternative splicing (PMID: 21177968), with on average, ~2 isoforms per transcript. Among our selected genes, we only had one case where a single tag would not capture all isoforms (*flad-1*). We examined an additional 30 random genes and found no more examples by chance. So, in our view, this will be rare though we recognize in some cases a practical decision will need to be made, which could involve consideration of expression levels of each terminal exon.

Finally, some readers might object to the authors' assertion in the abstract that this work is "a first step in this direction" (presumably referring to designing a strategy for whole-proteome tagging). There is no concern that the authors are disregarding the extensive work of other groups, as they explicitly mention the contributions of other groups to the foundation that enables the present work. However, the spirit of the abstract could be misinterpreted by a well-intentioned reader.

We appreciate the referee’s perspective and have reworded this phrase in the abstract to: “As proof-of-principle for scalable pooled tagging, we undertook a pilot study in the nematode *C. elegans*, in which we set out to tag 30 different genetic loci with three different fluorophores, with 3 tags being introduced at a time.”
